# Molecular and Histological Characterization of a Novel Hydrogel-Based Strategy for Inducing Experimental Glaucoma in Mice

**DOI:** 10.3390/ijms26209860

**Published:** 2025-10-10

**Authors:** Basel Obied, Stephen Richard, Judith Kramarz Dadon, Tal Corina Sela, Noa Geffen, Michal Halperin-Sternfeld, Lihi Adler-Abramovich, Nitza Goldenberg-Cohen, Alon Zahavi

**Affiliations:** 1 Department of Ophthalmology, Bnai-Zion Medical Center, Haifa 339419, Israel; basel.obied01@gmail.com (B.O.); ncohen1@gmail.com (N.G.-C.); 2 The Krieger Eye Research Laboratory, Ruth and Bruce Rappaport Faculty of Medicine, Technion—Institute of Technology, Haifa 3498838, Israel; steverit11@gmail.com; 3 Department of Ophthalmology and Eye Research Laboratory, Felsenstein Medical Research Center, Rabin Medical Center, Petach Tikva 4941492, Israel; jskramarz@gmail.com (J.K.D.); noatal1122@gmail.com (N.G.); 4 Gray Faculty of Medical and Health Sciences, Tel Aviv University, Tel Aviv 6997801, Israel; tal200390@gmail.com; 5 Department of Oral Biology, Goldschleger School of Dental Medicine, Gray Faculty of Medical and Health Sciences, Tel Aviv University, Tel Aviv 6997801, Israel; michal4@mail.tau.ac.il (M.H.-S.); lihia@tauex.tau.ac.il (L.A.-A.); 6 Department of Periodontology, Goldschleger School of Dental Medicine, Gray Faculty of Medical and Health Sciences, Tel Aviv University, Tel Aviv 6997801, Israel; 7 Jan Koum Center for Nanoscience and Nanotechnology, Tel Aviv University, Tel Aviv 6997801, Israel

**Keywords:** glaucoma model, mouse model, hyaluronic acid, self-assembling peptide, retinal ganglion cell loss

## Abstract

Glaucoma is a leading cause of irreversible blindness, and animal models are essential for studying its pathophysiology and testing therapeutic strategies. In this study, a novel hydrogel-based approach was developed and evaluated to induce experimental glaucoma in mice, using composites of hyaluronic acid (HA) and the self-assembling peptide fluorenylmethoxycarbonyl-diphenylalanine (FmocFF). Two formulations with different HA-to-FmocFF ratios were injected either intracamerally or intravitreally in C57BL/6 mice. Intraocular pressure (IOP) was monitored over 21 days, and retinal tissues were analyzed histologically and immunohistochemically. Significant IOP elevation was observed in one hydrogel formulation (Mixture B), yet without detectable retinal ganglion cell loss. A significant reduction in retinal ganglion cell (RGC) density, independent of IOP changes or injection site, was observed in Mixture A. Histological staining confirmed successful delivery and localization of the hydrogel in the anterior chamber, and no evidence of gliosis, microglial activation, or increased apoptosis was revealed by immunostaining. Collectively, these data position the HA-FmocFF hydrogel as a proof-of-concept that advances glaucoma model development, although it does not yet recapitulate the full disease. This model may facilitate future studies of neuroprotection and disease-modifying therapies in glaucoma without confounding inflammatory responses.

## 1. Introduction

Glaucoma is a progressive neurodegenerative disease of the optic nerve and a leading cause of irreversible blindness worldwide, posing a major public health challenge. Recent global estimates indicate that approximately 3.6 million people are blind and 4.1 million have moderate or severe visual impairment due to the disease [1,2]. Glaucoma accounts for nearly 8% of global blindness, underscoring its impact relative to other blinding conditions [3]. The worldwide prevalence is projected to exceed 110 million individuals by 2040 as populations age [4], emphasizing the urgent need for improved models to study disease mechanisms and evaluate potential therapies. Glaucoma is primarily driven by the loss of retinal ganglion cells (RGCs) and their axons [5,6,7]. While intraocular pressure (IOP) remains the most significant modifiable risk factor, disease progression often continues despite effective IOP-lowering interventions [8,9]. This underscores the urgent need for models that better capture both pressure-dependent and pressure-independent mechanisms of glaucomatous damage.

Animal models are indispensable for studying glaucoma pathogenesis and evaluating new therapeutic approaches [10]. Rodent models, in particular, offer advantages of cost, genetic tractability, and scalability [11,12]. Existing inducible models in mice and rats attempt to mimic human glaucoma via various strategies: microbead or viscoelastic occlusion of aqueous outflow, laser photocoagulation of trabecular meshwork or episcleral veins, episcleral vein cauterization, hypertonic saline injection, and genetic modifications [12,13,14,15]. However, these models often involve acute or inconsistent IOP elevation, require technically demanding procedures, or fail to replicate the chronic and progressive nature of glaucomatous optic neuropathy. Moreover, their reproducibility across laboratories remains a challenge.

Hydrogels have emerged as highly versatile biomaterials with broad biomedical applications, owing to their tunable physicochemical properties, high water content, and tissue-like mechanical characteristics. In drug delivery, hydrogels enable controlled, localized, and sustained release of small molecules, macromolecular drugs, proteins, nucleic acids, and even living cells, with established applications in oncology, immunology, wound healing, ophthalmology, and pain management [16,17,18]. In tissue engineering, they provide three-dimensional scaffolds that mimic the extracellular matrix, supporting cell proliferation, differentiation, and tissue formation, and have been successfully used to engineer liver, cartilage, cardiac, and skin tissues, as well as for stem cell and organoid cultures [19,20,21,22]. Hydrogel-based wound dressings maintain a moist healing environment, promote autolytic debridement, and serve as carriers for antibiotics or growth factors, making them valuable for treating burns, ulcers, and chronic wounds [23,24]. In ophthalmology, hydrogels are used as drug delivery vehicles and artificial vitreous substitutes [25,26,27]. Additional applications include matrices for biosensors, injectable therapies for minimally invasive drug depots or bulking agents, bioinks for 3D and 4D bioprinting of complex tissues, contact lenses with drug-delivery or sensing capacity, and bioadhesives or sealants for surgical and wound repair [21,28,29,30]. Collectively, these applications highlight hydrogels as a platform technology with broad potential across drug delivery, regenerative medicine, biosensing, soft robotics, and diagnostic fields, emphasizing their relevance for developing novel ocular disease models.

Recently, intracameral injection of cross-linked hydrogels has emerged as a promising strategy to induce ocular hypertension by mechanically obstructing aqueous humor outflow [31,32,33]. Previous studies have demonstrated the feasibility of this approach in rodents using chemically cross-linked hydrogels, resulting in transient IOP elevation and regional RGC loss. Nonetheless, many of these hydrogels suffer from poor in vivo stability and limited mechanical strength, especially those based solely on hyaluronic acid (HA), which is rapidly cleared from the anterior chamber [34].

Diphenylalanine (FF) is a well-characterized dipeptide widely used to create biocompatible ordered nanostructures [35,36]. Moreover, Fluorenylmethoxycarbonyl-diphenylalanine (FmocFF) spontaneously forms nanofibrous networks that entrap water, resulting in transparent, stable, and injectable hydrogels. These materials mimic the structural and mechanical properties of soft tissue and are highly tunable, allowing adjustment of stiffness and gelation speed to match different biomedical applications. Fluorenylmethoxycarbonyl-diphenylalanine (FmocFF) further stabilizes the hydrogel network, producing uniform and durable scaffolds [37,38]. Because of their biocompatibility and ability to support cell growth, FF-based hydrogels have been applied in drug delivery, tissue regeneration, and wound healing, making them promising candidates for developing controlled ocular disease models [39,40,41].

This study was aimed at developing and evaluating a novel hydrogel-based glaucoma model in mice. A self-assembling scaffold composed of HA and FmocFF was used. The feasibility of this composite scaffold, delivered via intracameral or intravitreal injection, was assessed with the aim of inducing sustained IOP elevation and RGC loss. By optimizing hydrogel composition, injection technique, and site of delivery, a reproducible and biocompatible model was sought to be established that could facilitate future studies of glaucoma pathophysiology and therapeutic intervention.

## 2. Results

Three mice, 2 from Group I and 1 from Group II, died within one hour of anesthesia administration. Additionally, one eye in Group II developed phthisis bulbi. These subjects were excluded from subsequent analysis.

### 2.1. IOP Outcomes

Baseline IOP values were similar to those reported in prior studies [42,43]. Throughout the experimental period, no significant IOP increase was demonstrated in eyes injected with Mixture A compared to baseline, regardless of injection site (Table 1).

**Figure 1:**
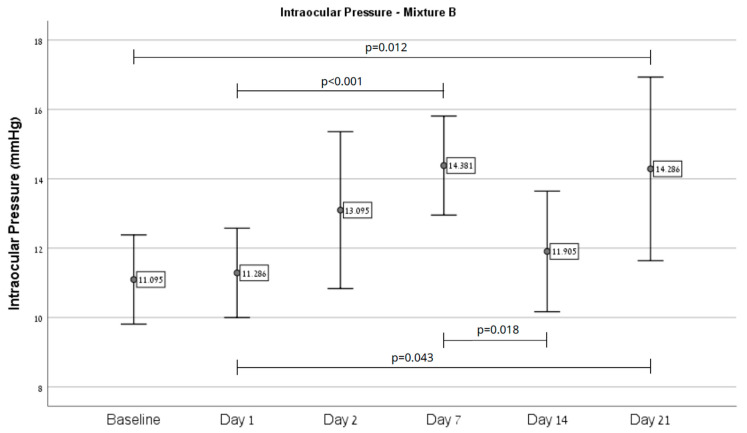
A significant difference in IOP over time was demonstrated in eyes injected with Mixture B. Post hoc pairwise comparison showed significant differences in IOP between baseline and day 21 (*p* = 0.012), between days 1 and 7 (*p* < 0.001), between days 1 and 21 (*p* = 0.043), and between days 7 and 14 (*p* = 0.018).

**Figure 2:**
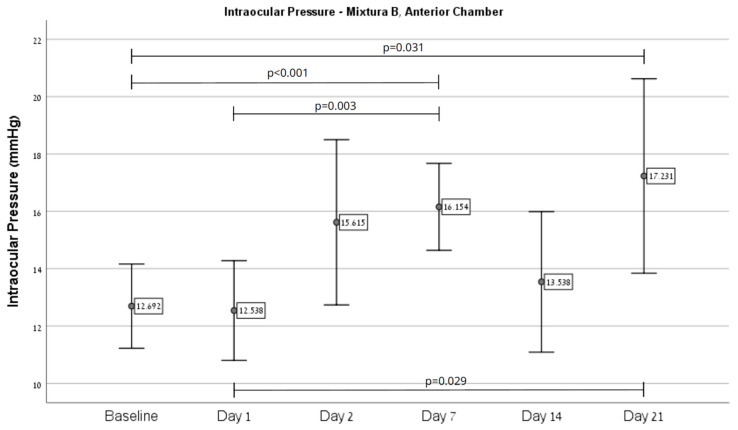
A significant difference in IOP over time was demonstrated in eyes injected with Mixture B to the anterior chamber. Post hoc pairwise comparison showed significant differences in IOP between baseline and day 7 (*p* < 0.001), baseline and day 21 (*p* = 0.031), between days 1 and 7 (*p* = 0.003), and between days 1 and 21 (*p* = 0.029).

**Figure 3:**
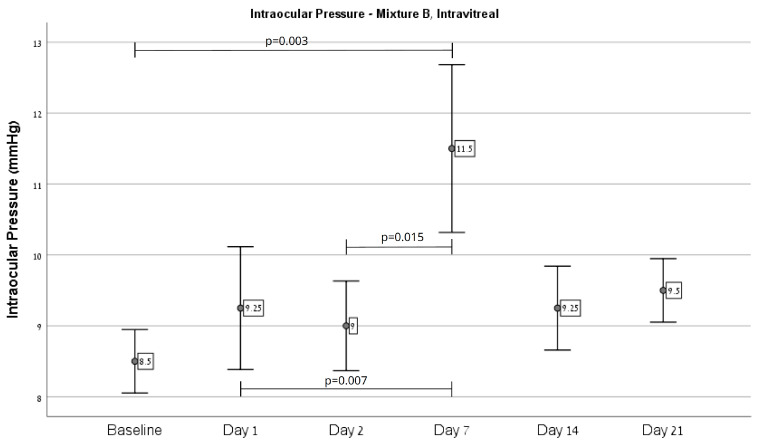
A significant difference in IOP over time was demonstrated in mice eyes injected with Mixture B to the vitreous. Post hoc pairwise comparison showed significant differences in IOP between baseline and day 7 (*p* = 0.003), between days 1 and 7 (*p* = 0.007), and between days 2 and 7 (*p* = 0.015).

Overall, an increase in IOP was observed in eyes injected with Mixture B (*n* = 21) from baseline (11.0 ± 2.83) on day 21 (14.29 ± 5.81, *p* = 0.012), as depicted in Figure 1. Regarding injection site effects, Mixture B intracameral injections (*n* = 13) showed an increase from baseline (12.69 ± 2.43) on day 7 (16.15 ± 2.51, *p* < 0.001) and 21 (17.23 ± 5.61, *p* = 0.031), as depicted in Figure 2. Injection to the vitreous (*n* = 8) demonstrated an increase from baseline (8.5 ± 0.53) on day 7 (11.5 ± 1.41, *p* = 0.003), as depicted in Figure 3.

### 2.2. RGC Survival

A notable impact of hydrogel composition on RGC preservation was observed. Mixture A was associated with a significant reduction in RGC density (22.0 ± 4.0 cells per 300 μm) compared to controls (*p* < 0.001), regardless of the injection site. In contrast, Mixture B yielded a mean RGC count of 26.2 ± 4.7, which was not significantly different from control eyes.

When stratified by injection site, no significant differences in RGC counts were observed. Intracameral injections resulted in an average of 22.4 ± 4.3 cells per 300 μm, while intravitreal injections yielded 23.6 ± 4.7 (*p* = 0.260 vs. controls).

IOP did not significantly influence RGC survival, as RGC counts were comparable between eyes with elevated IOP and those without. The mean RGC count in eyes with high IOP was 34.3 ± 3.7, compared to 36.1 ± 4.6 in eyes with normal IOP (*p* = 0.243).

### 2.3. Immunohistochemistry

**Figure 4:**
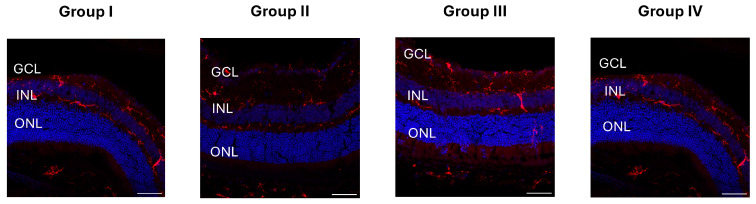
Iba1 immunostaining of retinal sections. Iba1 staining (red) revealed no significant differences in microglial distribution or morphology across experimental groups, indicating that none of the injection conditions (hydrogel composition or injection site) induced detectable retinal inflammation. Nuclei were counterstained with DAPI (blue). Group I: intracameral injection of Mixture A; Group II: intravitreal injection of Mixture A; Group III: intracameral injection of Mixture B; Group IV: intravitreal injection of Mixture B. Representative merged images are shown for each group. Scale bar = 50 μm.

To investigate potential cellular responses to hydrogel-induced IOP elevation, retinal sections were immunostained for IBA1, glial fibrillary acidic protein (GFAP), NeuN, and TUNEL. IBA1, a marker of microglia, did not show evidence of activation, such as changes in morphology or increased soma size, in any experimental group compared to controls (Figure 4).

**Figure 5:**
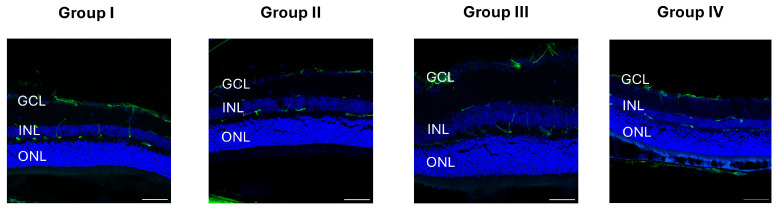
GFAP immunostaining of retinal sections. GFAP staining (green) showed no significant differences in astrocytic or Müller cell reactivity across experimental groups, suggesting that none of the injection conditions (hydrogel composition or injection site) induced retinal gliosis. Nuclei were counterstained with DAPI (blue). Group I: intracameral injection of Mixture A; Group II: intravitreal injection of Mixture A; Group III: intracameral injection of Mixture B; Group IV: intravitreal injection of Mixture B. Representative merged images are shown for each group. Scale bar = 50 μm.

GFAP, which indicates reactive gliosis in Müller cells and astrocytes, also showed no upregulation or redistribution consistent with a gliotic response (Figure 5).

**Figure 6:**
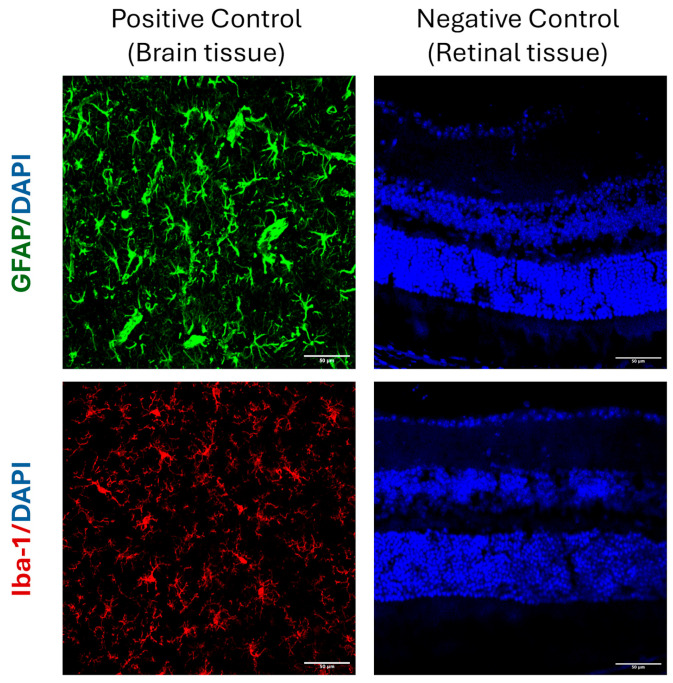
Positive and negative controls for immunofluorescence staining. Representative images demonstrating antibody specificity for GFAP (green) and IBA-1 (red). Brain tissue served as a positive control and shows robust astrocytic and microglial immunoreactivity. Retinal sections processed in parallel with omission of primary antibodies served as negative controls and exhibited no detectable signal, confirming the absence of nonspecific staining. Nuclei were counterstained with DAPI (blue). Scale bar = 50 µm.

To confirm antibody specificity and rule out nonspecific background staining, negative controls were processed in parallel with omission of the primary antibodies while all other staining steps were performed identically. These controls showed no detectable signal, confirming that the observed immunoreactivity reflected true antigen–antibody binding. Brain tissue served as a positive control and demonstrated strong GFAP and IBA-1 labeling. Representative positive and negative controls are shown in Figure 6.

NeuN, a neuronal marker that predominantly labels RGCs, revealed preserved RGC architecture without significant loss or redistribution among the different groups. TUNEL staining, used to detect apoptotic cells, demonstrated similar numbers of positive cells across all groups, with no indication of increased apoptosis following hydrogel injection or IOP elevation.

Taken together, these findings suggest that despite the patterns of IOP elevation induced in this study, there was no detectable neuroinflammatory or gliotic response, and RGC structural integrity was preserved, as assessed by NeuN immunostaining. Histological evaluation confirmed that intracameral hydrogel placement did not cause detectable damage to the trabecular meshwork, corneal endothelium, or lens structures, supporting the safety of the injection procedure.

## 3. Discussion

Intraocular cross-linked hydrogel injection has emerged as a promising strategy for inducing mild to moderate ocular hypertension in experimental models, with the goal of mimicking features of human glaucoma [44,45]. In the present study, a reliable model of IOP elevation was established using a novel hydrogel formulation composed of hyaluronic acid (HA) in combination with FmocFF, a low-molecular-weight peptide gelator known for forming stable hydrogels [38,41,46]. It was hypothesized that modifying the hydrogel composition and the injection site might induce sustained IOP elevation, ultimately producing significant RGC loss and neuroinflammatory changes comparable to human glaucoma pathophysiology [47,48].

**Figure 7:**
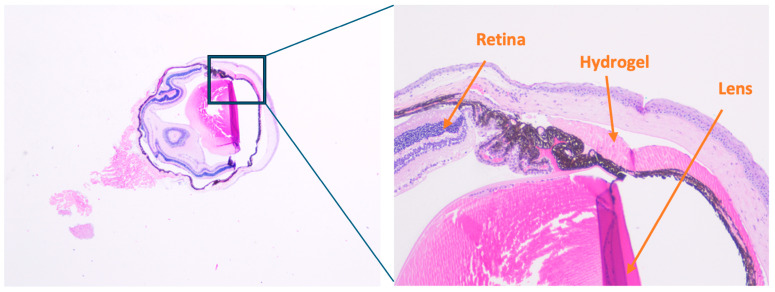
Localization of hydrogel following intracameral injection. Hematoxylin and eosin (H&E) staining shows the presence of hydrogel (Mixture A) at the anterior chamber angle immediately after a single intracameral injection. The zoomed-in image highlights the hydrogel material situated between the cornea and lens, adjacent to the trabecular meshwork. Retinal and lens structures are identifiable, confirming proper anatomical orientation and successful delivery of the hydrogel to the target site. Minor sectioning artifacts, including gaps and tissue folds, are present but do not interfere with interpretation of the key anatomical features. Histological assessment was performed on multiple sections from each eye to ensure reliability of the observations.

While an IOP increase was demonstrated following injection of Mixture B, RGC loss was observed only after Mixture A injections. Despite this discrepancy, several important insights and key strengths were demonstrated in this study. First, two novel hydrogel formulations composed of HA and FmocFF were successfully synthesized and delivered. This validated the feasibility and reproducibility of the injection procedure using both intracameral and intravitreal routes in a murine model. Second, IOP elevation was achieved in Mixture B, while a statistically significant reduction in RGC density was consistently induced by Mixture A, suggesting a direct effect on retinal ganglion cell integrity independent of measurable pressure elevation. Third, histological sections demonstrated the presence of the hydrogel mixture in the anterior chamber following injection, despite undergoing standard chemical staining procedures (Figure 7). This histological confirmation of hydrogel presence in the anterior chamber supports the reliability of the injection procedure. Fourth, no evidence of gliosis, neuroinflammation, or apoptosis was revealed by immunohistochemical analyses in any of the experimental groups, supporting the biocompatibility of the hydrogel materials. Importantly, no histological abnormalities or retinal toxicity attributable to DMSO were observed in any group, supporting the safety of its use at the very low final concentration employed (2.5%). Collectively, these findings underscore the platform’s potential as a basis for further optimization, while highlighting the model’s suitability for investigating pressure-independent mechanisms of RGC loss and evaluating neuroprotective strategies without confounding inflammatory responses.

HA is widely used in ophthalmic procedures due to its biocompatibility, but its rapid clearance and poor mechanical stability limit its use as a long-acting obstructive agent within the anterior chamber [49]. Previous reports have demonstrated that soluble HA fails to sustain IOP elevation beyond 24 h following intracameral injection, primarily because it is rapidly removed through physiological outflow pathways [50]. Hydrogel persistence in the anterior chamber was intended to be prolonged by formulating HA chains with FmocFF in the anterior chamber, anticipating that this would better obstruct aqueous outflow at the trabecular meshwork. FmocFF self-assembles at physiological pH and higher temperatures, forming a transparent, in situ hydrogel that can be delivered in liquid form at room temperature but rapidly gels after injection into the eye [51].

Two hydrogel formulations differing in their relative proportions of FmocFF and hyaluronic acid were tested: Mixture A, with a higher FmocFF-to-HA ratio, was designed to increase the gel’s mechanical stiffness and resistance to deformation, while Mixture B, with a lower FmocFF-to-HA ratio, was intended to enhance injectability and flow properties. The rationale was that a stiffer gel (Mixture A) might better obstruct the trabecular meshwork, while a more fluid formulation (Mixture B) could more uniformly distribute within the anterior chamber angle. Successful delivery of both mixtures to the anterior chamber and vitreous cavity was achieved. However, only Mixture B resulted in a measurable IOP increase, whereas Mixture A produced minimal or no pressure elevation. This likely reflects differences in gel consistency and behavior within the anterior chamber, with Mixture A possibly dispersing or degrading more rapidly and therefore exerting less effect on aqueous outflow resistance.

A clear relationship was observed between hydrogel composition and the degree of IOP elevation. Mixture B, which contained a lower FmocFF-to-HA ratio, produced a more pronounced and sustained IOP rise compared with Mixture A. This effect may reflect Mixture B’s greater fluidity and uniform distribution within the anterior chamber angle, leading to more effective obstruction of aqueous outflow. In contrast, the stiffer Mixture A likely failed to form a continuous angle-blocking plug or was displaced by aqueous humor dynamics, resulting in negligible IOP elevation. These findings underscore the importance of tuning the mechanical and rheological properties of peptide–HA hydrogels to achieve reproducible and physiologically relevant IOP elevation in experimental models. Future studies should systematically vary the HA and FmocFF ratios and assess additional rheological parameters to identify the optimal formulation for sustained and controlled IOP elevation suitable for long-term glaucoma research [52].

Although Mixture B produced a statistically significant IOP rise, it did not translate into detectable RGC loss, likely because the magnitude and/or duration of elevation was below the injury threshold. The discordance between the observed RGC loss following injection of Mixture A and the absence of detectable apoptosis, gliosis, or microglial activation raises important considerations. One plausible explanation is that Mixture A exerts a direct or delayed toxic effect on RGCs, independent of pressure elevation or classical inflammatory pathways. Although the hydrogel components were selected based on prior evidence of biocompatibility, it is possible that the higher FmocFF content or altered physical properties (e.g., stiffness, degradation kinetics, local pH) contributed to subclinical toxicity not captured by our acute histological markers. Alternatively, the model may reflect a chronic degenerative process wherein initial sublethal stress leads to delayed RGC loss without a prominent apoptotic peak. The TUNEL assay, while sensitive for detecting acute DNA fragmentation, may fail to detect low-grade or asynchronous cell death occurring over a prolonged period. Similarly, absence of GFAP or IBA1 upregulation does not exclude subtle or localized glial changes below the detection threshold. Future studies incorporating longitudinal analysis, ultrastructural evaluation, and transcriptomic profiling may help clarify the mechanism of RGC loss and distinguish between mechanical, toxic, and pressure-independent neurodegenerative pathways.

Beyond testing hydrogel composition, we also investigated two distinct injection sites to explore alternative mechanisms of IOP elevation. Intracameral injection was intended to directly obstruct aqueous outflow by blocking the trabecular meshwork, thereby elevating IOP through reduced drainage. In contrast, intravitreal injection aimed to induce a relative pupillary block by displacing the lens-iris diaphragm anteriorly, theoretically impeding aqueous humor passage from the posterior to the anterior chamber. Despite these targeted approaches, neither injection site produced a significant or sustained elevation of IOP in our model. This outcome is consistent with previous reports indicating that pupillary block mechanisms are difficult to reliably replicate in mice, likely due to anatomical differences in the anterior segment as well as distinct aqueous humor dynamics compared to the human eye [12,53].

Finally, no significant neuroinflammatory or neurodegenerative changes were observed in retinal sections, as indicated by the absence of increased IBA1 or GFAP immunoreactivity, preserved NeuN-positive RGC layers, and comparable TUNEL staining among groups. This further suggests that neither the hydrogel itself nor the minimal IOP changes induced were sufficient to trigger secondary retinal gliosis or neurodegeneration.

Our study had certain limitations. The variability in hydrogel properties, and the lack of in vivo confirmation of gel stability within the anterior chamber, may have contributed to the failure to induce chronic IOP elevation in Mixture A, and a clinically significant IOP rise in Mixture B sufficient to result in RGC loss. Future refinements could include higher peptide concentration, different delivery vehicles, or adjunctive methods to maintain hydrogel positioning within the chamber angle. In addition, another limitation of this study is the absence of a direct comparison with an established glaucoma model, such as microbead-induced glaucoma. Although our data demonstrate successful hydrogel localization and no detectable retinal toxicity for either Mixture A or Mixture B, future studies should incorporate these conventional models to enable head-to-head comparisons and better contextualize the translational relevance of the hydrogel approach. Finally, larger animal models with more comparable aqueous dynamics to human eyes may improve the translational potential of hydrogel-induced glaucoma models [54,55].

This study was designed as a proof-of-concept to characterize a novel hydrogel-based approach for controlled IOP elevation, focusing on demonstrating hydrogel localization, transient IOP rise, and the absence of acute retinal toxicity. We acknowledge that glaucoma pathophysiology is multifactorial and develops over time, with progressive retinal ganglion cell loss, glial activation, and visual dysfunction typically requiring sustained IOP elevation and prolonged follow-up to observe. Future studies will incorporate extended observation periods and functional assessments, including RGC counts, GFAP quantification, and functional studies, as well as direct comparisons with established glaucoma models. These efforts will be essential to fully validate this hydrogel-based strategy as a reliable and translational model for glaucoma research.

## 4. Materials and Methods

### 4.1. Study Animals

Seventy wild-type (WT) C57BL/6 mice (age 4 to 6 weeks, mean weight 28 g) were included in the study. Mice were obtained from Envigo Laboratories (Jerusalem, Israel). Study animals were maintained and handled in accordance with the Association for Research in Vision and Ophthalmology (ARVO) Statement for the Use of Animals in Ophthalmic and Vision Research and the National Institutes of Health guidelines. All animal experiments were conducted in accordance with institutional guidelines and approved by the Rabin Medical Center Institutional Animal Research Committee (approval code: b18314_4_022 051120).

### 4.2. Hydrogel-Induced Glaucoma Model

Two hydrogel formulations were prepared for intraocular injection. First, lyophilized FmocFF peptide powder (Bachem, Budendorf, Switzerland) was dissolved in dimethyl sulfoxide (DMSO) at a concentration of 150 mg/mL. High molecular weight (3 × 10^6^) hyaluronic acid as sodium salt of 1% HA in PBS in syringes (BTGFerring, Kiryat Malachy, Israel) were diluted in ddH_2_O, resulting in a final concentration of 0.5%. Then, the hydrogels were formed by mixing the FmoFF stock solution with the HA solution using vortex to create two mixtures: Mixture A (*n* = 45) consisted of FmocFF and HA at a 3:1 ratio peptide to HA (3.75 mg FmocFF and 1.25 mg HA in 1 mL solution). Mixture B (*n* = 25) was prepared by mixing FmocFF and HA at a 1:3 ratio (1.25 mg FmocFF and 3.75 mg HA in 1 mL solution).

Each mixture was injected into either the anterior chamber or vitreous of the right eye under general anesthesia with ketamine (80 mg/kg) and xylazine (4 mg/kg), supplemented with topical proparacaine hydrochloride 0.5% for local anesthesia.

### 4.3. Measurement of IOP

**Figure 8:**
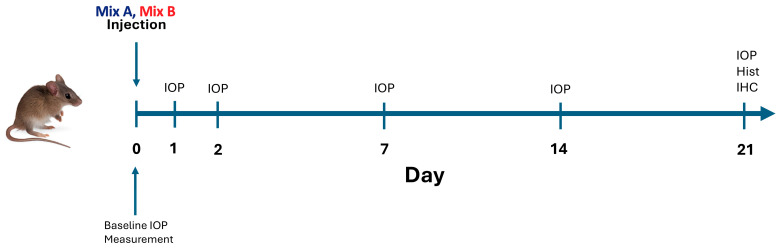
Study Timeline. Intraocular pressure (IOP) was measured on days 0, 1, 2, 7, 14, and 21 following hydrogel injection. On day 21, mice were euthanized via CO_2_ asphyxiation, and both globes and optic nerves were enucleated for subsequent histological and immunohistochemical analyses. Hist- Histology, IHC—Immunohistochemistry.

IOP measurements were performed using a rebound tonometer calibrated for mice (Icare^®^ TonoLab, Vantaa, Finland). Measurements were taken at baseline (prior to intervention) and on days 1, 2, 7, 14, and 21 following hydrogel injection. Figure 8 presents the study design and timeline. All measurements were performed under anesthesia and repeated three times per eye. The average of the three readings was calculated and recorded.

### 4.4. Experimental Groups and Study Design

The mice were divided into four groups depending on mixture type and injection site (anterior chamber versus intravitreal). A volume of 3 μL hydrogel of Mixture A or Mixture B was injected intravitreally or intracamerally as per group allocation.

Group I (*n* = 40) received a single intracameral injection of Mixture A into the right eye, while Group II (*n* = 5) received an intravitreal injection of the same mixture. Group III (*n* = 16) received a single intracameral injection of Mixture B, while Group IV (*n* = 9) received an intravitreal injection of the same mixture.

### 4.5. Histological Analysis

Following enucleation, eyes were fixated in 4% formaldehyde for 1 h, then washed in phosphate-buffered saline (PBS, 1X; Beit HaEmek, Israel). Samples underwent sequential incubation in 15% and 20% sucrose solutions (in PBS) for 1 h each, followed by immersion in 30% sucrose at 4 °C for 12 h. Eyes were then embedded in optimal cutting temperature (O.C.T) compound and stored at −80 °C for 24 h. Cryosections were prepared at 10 μm thickness using Leica CM1850 Cryostat (Leica, Buffalo Grove, IL, USA).

### 4.6. Hematoxylin and Eosin

Sagittal sections (10 μm) of each eye were stained with hematoxylin and eosin. Four consecutive sections were mounted on each slide and examined under a light microscope (Ernst Leitz GMBH, Wetzlar, Germany).

### 4.7. TUNEL Immunostaining

TdT-mediated dUTP Nick End-Labeling (TUNEL) assay staining was performed according to the manufacturer’s instructions (Roche Diagnostics GmbH, Mannheim, Germany). The sections underwent nuclear counterstaining with DAPI. Results were analyzed with a confocal fluorescence microscope (LSM 700 Inverted) equipped with appropriate filters. Excitation wavelengths used were 405 nm for DAPI and 488 nm for Cy2. The mean number of TUNEL-positive cells was determined in five different regions in each section, and plotted as a column chart with standard deviation.

### 4.8. GFAP, NeuN, and Iba1 Immunostaining

Retinal and optic nerve sections were placed on slides and washed with PBS prior to blocking with 5% fetal calf serum and 1% Triton X-100 for 1 h. The sections were then incubated with the primary anti- ionized calcium-binding adapter molecule 1(Iba-1) (1:500, abcam), anti-NeuN (1:500, Merck Millipore, Burlington, MA, USA), and anti-GFAP (1:500, abcam), at 4 °C overnight. The slides were incubated with the secondary antibodies, goat anti-rabbit Alexa fluor 647 (diluted 1:1000, abcam) and goat anti-chicken IgG NL-577 (diluted 1:200, R&D Systems-Biotest, Minneapolis, MN, USA), at room temperature for 1 h. The retinal sections were counterstained with 4′, 6-diamidino-2-phenylinodole (DAPI) (Molecular Probes Invitrogen, Eugene, OR, USA) to reveal cell nuclei. Images were obtained using a confocal fluorescence microscope (Zeiss LSM700, Munchen, Germany).

### 4.9. RGC Counts

RGCs were counted in the midperipheral retina, approximately 300 μm from the optic disk. Cell counts were performed on 6 sections per globe. In each section, a single row of RCGs was quantified within a 20× magnification field. The mean number of cells per field was then calculated.

## 5. Conclusions

In conclusion, this proof-of-concept study demonstrated that the HA–FmocFF hydrogel system can be successfully delivered to the anterior chamber and vitreous, producing a transient IOP elevation without causing detectable retinal toxicity, gliosis, or apoptosis. Mixture B, with a lower FmocFF-to-HA ratio, achieved a more consistent IOP rise compared with Mixture A, underscoring the importance of tuning hydrogel composition to optimize pressure elevation. Although significant RGC loss was not observed under the conditions tested, these findings provide valuable insight into hydrogel behavior in vivo and inform strategies for further refinement [51,56,57,58]. Future studies should further optimize hydrogel stiffness, degradation kinetics, and injection volume to achieve reproducible, sustained IOP elevation that induces progressive RGC loss. Incorporating established glaucoma models, extended follow-up, and functional assessments will be essential to validate this approach as a translationally relevant glaucoma model. Ultimately, a reproducible and physiologically meaningful hydrogel-based model may serve as a powerful platform to investigate pressure-dependent and pressure-independent neurodegenerative mechanisms and to evaluate candidate neuroprotective or disease-modifying therapies.

## Figures and Tables

**Table 1:** Intraocular pressure (IOP, mmHg) over 21 days following intracameral or intravitreal injection of HA–FmocFF hydrogels. Values are mean ± SD at baseline (pre-injection) and days 1, 2, 7, 14, and 21, shown separately for Mixture A and Mixture B by injection site and pooled across sites. Mixture B exhibited a significant increase in IOP over time, with higher IOP versus baseline on day 7 and day 21 overall and within both routes (overall *p* < 0.001; intracameral *p* < 0.001; intravitreal *p* = 0.001). Mixture A showed no significant IOP change. Abbreviations: IOP, intraocular pressure; SD, standard deviation.

IOP (mmHg)	Mixture A						Mixture B					
	Anterior Chamber		Intravitreal		All Eyes		Anterior Chamber		Intravitreal		All Eyes	
	Mean	SD	Mean	SD	Mean	SD	Mean	SD	Mean	SD	Mean	SD
Baseline	10.96	1.94	9.25	0.50	10.70	1.90	12.69	2.43	8.50	0.53	11.10	2.83
Day 1	8.09	0.95	13.00	10.68	8.81	4.13	12.54	2.88	9.25	1.04	11.29	2.83
Day 2	11.57	3.80	11.50	5.07	11.56	3.90	15.62	4.77	9.00	0.76	13.10	4.97
Day 7	12.35	2.77	21.00	16.75	13.63	6.98	16.15	2.51	11.50	1.41	14.38	3.14
Day 14	11.83	3.47	11.25	5.25	11.74	3.66	13.54	4.05	9.25	0.71	11.90	3.82
Day 21	11.61	3.60	11.75	4.19	11.63	3.61	17.23	5.61	9.50	0.53	14.29	5.82

## Data Availability

The datasets generated during and/or analyzed during the current study are available from the corresponding author upon reasonable request.

## Abbreviations

The following abbreviations are used in this manuscript:

HA	Hyaluronic acid
FmocFF	Fluorenylmethoxycarbonyl-diphenylalanine
IOP	Intraocular pressure
RGC	Retinal ganglion cell
GFAP	Glial fibrillary acidic protein
